# A Greener Stability-Indicating High-Performance Thin-Layer Chromatography Approach for the Estimation of Topiramate

**DOI:** 10.3390/ma15051731

**Published:** 2022-02-25

**Authors:** Mohammed H. Alqarni, Faiyaz Shakeel, Wael A. Mahdi, Ahmed I. Foudah, Tariq M. Aljarba, Sultan Alshehri, Mohammed M. Ghoneim, Prawez Alam

**Affiliations:** 1 Department of Pharmacognosy, College of Pharmacy, Prince Sattam Bin Abdulaziz University, Al-Kharj 11942, Saudi Arabia; m.alqarni@psau.edu.sa (M.H.A.); a.foudah@psau.edu.sa (A.I.F.); t.aljarba@psau.edu.sa (T.M.A.); 2 Department of Pharmaceutics, College of Pharmacy, King Saud University, Riyadh 11451, Saudi Arabia; faiyazs@fastmail.fm (F.S.); wmahdi@ksu.edu.sa (W.A.M.); salshehri1@ksu.edu.sa (S.A.); 3 Department of Pharmacy Practice, College of Pharmacy, AlMaarefa University, Ad Diriyah 13713, Saudi Arabia; mghoneim@mcst.edu.sa

**Keywords:** AGREE score, greener HPTLC, topiramate, validation

## Abstract

Despite various reported analytical methods for topiramate (TPM) analysis, greener analytical approaches are scarce in literature. As a consequence, the objective of the current research is to design a normal-phase stability-indicating high-performance thin-layer chromatography (SI-HPTLC) methodology for TPM analysis in marketed tablet dosage forms that is rapid, sensitive, and greener. TPM was derivatized densitometrically and analyzed at 423 nm in visible mode with anisaldehyde-sulfuric acid as the derivatizing agent. The greener SI-HPTLC technique was linear in the 30–1200 ng band^−1^ range. In addition, the suggested SI-HPTLC methodology for TPM analysis was simple, rapid, cheaper, precise, robust, sensitive, and environmentally friendly. The greener SI-HPTLC method was able to detect TPM along with its degradation products under acid, base, and oxidative degradation conditions. However, no TPM degradation was recorded under thermal and photolytic stress conditions. TPM contents in commercial tablet dosage forms were recorded as 99.14%. Using 12 different principles of green analytical chemistry, the overall analytical GREEnness (AGREE) score for the greener SI-HPTLC method was calculated to be 0.76, confirming the proposed normal-phase SI-HPTLC method’s good greener nature. Overall, these results demonstrated that the suggested SI-HPTLC technique for TPM measurement in pharmaceutical products was reliable and selective.

## 1. Introduction

Topiramate (TPM) is a sulfamate-substituted derivative of the monosaccharide D-fructose, which is used as an antiepileptic drug in the treatment of different kinds of seizures and epileptic disorders [1]. It acts on the central nervous system by blocking voltage-sensitive sodium channels and increasing the activity of γ-amino butyric acid [2,3]. Regular analysis of TPM is not easy because it does not have ultra-violet (UV), visible, or fluorescence absorption [4]. Therefore, the analysis of TPM is possible via derivatization using different derivatizing agents. Therefore, qualitative and quantitative analyses of TPM are necessary in order to determine it in the variety of pharmaceutical and biological samples.

Various pharmaceutical assays have been reported for the determination of TPM in different dosage forms and physiological fluids, such as plasma, serum, blood, and human breast milk. A spectrofluorimetry assay was used for TPM analysis in combination with levetiracetam in tablet dosage forms and human plasma samples [5]. The analysis of TPM using the specrofluorimetry method was possible via derivatization using a 4-chloro-7-nitrobenzofuran-2-oxo-1,3-diazole (NBD-Cl) derivatizing agent [5]. A colorimetry method was also utilized for TPM analysis in its pure form and tablet dosage forms [6]. Various high-performance liquid chromatography (HPLC) methods have been utilized for the determination of TPM in its pure/bulk form and different dosage forms either utilizing special kinds of detectors or using derivatizing agents [4,7,8,9,10,11]. A high-performance thin-layer chromatography (HPTLC) method was utilized for the determination of TPM in dosage forms, solubility study samples, diffusion study samples, plasma samples, and brain homogenates using the derivatizing agent as the visualizing agent [12]. The HPTLC method was also used for the evaluation of TPM degradation products [13]. The HPTLC method has also been used for TPM analysis in pharmaceutical formulations [14]. Additionally, the HPTLC method has been established for the detection of TPM in human serum samples and its further application to therapeutic drug monitoring [15]. For the detection of TPM in human breast milk samples, an HPTLC assay has been developed [16]. The liquid chromatography tandem mass spectrometry (LC-MS) and ultra-performance liquid chromatography (UPLC) methods have also been documented for TPM analysis in its bulk form and dosage forms [17,18]. Other approaches for determining TPM in its formulations have also been published, including flow-injection spectrometry, nuclear magnetic resonance (NMR) spectrometry, and gas-chromatography mass-spectrometry (GC-MS) tests [19,20,21]. TPM in plasma and serum samples has also been determined using HPLC-based techniques [22,23]. Various LC-MS assays have been documented for TPM analysis in human plasma samples [24,25,26,27]. TPM analysis in human hair samples was also done using the LC-MS method [28]. A liquid chromatography diode array detection (LC-DAD) method was utilized for TPM analysis in human serum and umbilical cord blood samples [29]. Various GC-MS techniques were applied for TPM analysis in human dried blood spots, serum, and plasma samples [30,31,32]. Some other techniques, such as capillary electrophoresis and chemosensor techniques, were used for TPM analysis in human plasma samples [33,34].

We discovered that the safety and greener properties of literature analysis procedures were not assessed after evaluating TPM estimation methods in the literature. Furthermore, the greener or environmentally friendly HPTLC methods for TPM analysis in commercial formulations and physiological fluids have yet to be described. Greener HPTLC methods present several advantages, such as “simplicity, economicity, low operation cost, short analysis time, parallel analysis of multiple samples, detection clarity, and reduction in environmental toxicity” compared to other liquid chromatography-based analytical techniques [35,36,37,38]. Therefore, a normal-phase stability-indicating HPTLC (SI-HPTLC) technique for TPM analysis was used in this research. For the evaluation of greener aspects of analytical procedures, many environmentally friendly methodologies are used [37,38,39,40,41,42]. However, the analytical GREEnness (AGREE) method exclusively utilizes all 12 principles/components of green analytical chemistry (GAC) for the evaluation of greener profiles of analytical methods [41]. Therefore, the AGREE metric approach was applied to evaluate the proposed normal-phase SI-HPTLC assay’s greener profiles [41]. According to the GAC principle, the examined solvents, such as cyclohexane (CyHex) and ethyl acetate (EtAc), are classified as green solvents [43,44,45]. Due to their non-toxicity towards the environment, these solvents are considered green solvents [45,46]. As a consequence, the binary combination of CyHex and EtAc was used as the greener mobile phase in this study. Based on all of these ideas, the current research aims to design and validate a rapid, sensitive, and greener normal-phase SI-HPTLC technique for the detection of TPM in marketed tablet dosage forms. The greener profiles of the proposed densitometric assay were evaluated by “AGREE: The Analytical Greenness Calculator”. The greener normal-phase SI-HPTLC technique for TPM estimation was validated by following the “International Council for Harmonization (ICH) Q2 (R1)” recommendations [47].

## 2. Materials and Methods

### 2.1. Materials

The standard TPM (purity: 99.3%) was obtained as a kind gift sample from Riyadh Pharmaceuticals (Riyadh, Saudi Arabia). The chromatography-grade CyHex and EtAc were procured from E-Merck (Darmstadt, Germany). Other materials and reagents were of analytical grades. Marketed tablet dosage forms of TPM were purchased from a local pharmacy shop in Riyadh, Saudi Arabia.

### 2.2. Chromatography and Analysis

The normal-phase HPTLC analysis of TPM in its pure/bulk form and commercial tablets was carried out using a “CAMAG HPTLC instrument (CAMAG, Muttenz, Switzerland)”. The detection of TPM in normal-phase state was conducted on “10 × 20 cm^2^ aluminum plates pre-coated with normal-phase silica gel 60 F254S plates (E-Merck, Darmstadt, Germany)”. The solutions to the normal-phase TLC plates were applied as the 6 mm bands using a “CAMAG Automatic Sampler 4 (ATS4) applicator (CAMAG, Geneva, Switzerland)”. The applicator for sampling was fitted to the “CAMAG Microliter Syringe (Hamilton, Bonaduz, Switzerland)”. The application rate for the determination of TPM in normal-phase state was fixed at 150 nL s^−1^. The normal-phase TLC plates were established in an “Automatic Developing Chamber 2 (ADC 2) (CAMAG, Muttenz, Switzerland)” at a distance of 80 mm. The greener mobile phase for the determination of TPM was CyHex-EtAc (40:60, *v v*^−1^). The development chamber was saturated previously with the vapors of CyHex-EtAc (40:60, *v v*^−1^) for 30 min at 22 °C. The scanning rate was kept constant at 20 mm s^−1^, and the slit dimensions were fixed at 4 × 0.45 mm^2^.

### 2.3. Derivatization and Scanning

The plates were viewed under the CAMAG UV cabinet after generating normal-phase TLC plates in the ADC 2 chamber. The compound TPM is not UV active, so the developed normal-phase CAMAG glass reagent spray technique was used to derivatize TLC plates by spraying them with anisaldehyde-sulfuric acid reagent. After derivatization with anisaldehyde-sulfuric acid reagent, the spots of TPM were visible. The normal-phase TLC plates were then heated at 110 °C for 10 min. TPM was determined after 30 min by scanning the plates at 423 nm with a “CAMAG TLC scanner III coupled to the WinCAT’s (v. 1.2.3., CAMAG, Muttenz, Switzerland)” software. Each analysis was done in at least three copies (n = 3).

### 2.4. Preparation of TPM Standard Solutions for Calibration and Quality Control (QC)

The required quantity of TPM (10 mg) was dissolved in 100 mL of CyHex-EtAc (40:60, *v v^−1^*) greener mobile phase to obtain a stock solution of TPM with a concentration of 100 µg mL^−1^. To generate TPM concentrations in the 30–1200 ng band^−1^ range, various amounts of this stock solution were diluted again with CyHex-EtAc (40:60, *v v*^−1^) greener mobile phase. TPM solutions were made at various concentrations and spotted to TLC plates. The TLC response for TPM was observed for each TPM concentration utilizing the suggested HPTLC assay. To obtain a TPM calibration plot, TPM concentrations were plotted against measured TLC response. In addition, three QC samples were created separately for the evaluation of different parameters for the suggested SI-HPTLC method, including low QC (LQC; 100 ng band^−1^), middle QC (MQC; 400 ng band^−1^), and high QC (HQC; 1200 ng band^−1^).

### 2.5. Validation Studies

The suggested analytical method for TPM analysis was validated for a variety of parameters, as per ICH-Q2-R1 recommendations [47]. Graphing TPM concentrations vs. measured TLC response was used to test TPM linearity. For the greener normal-phase SI-HPTLC assay, TPM linearity was tested at nine different QC solutions of 30, 60, 100, 200, 300, 400, 500, 600, and 1200 ng band^−1^. “Retardation factor (R_f_), asymmetry factor (As), and number of theoretical plates per meter (N m^−1^)” were used to determine system suitability/efficiency for the proposed analytical test. The R_f_, As, and N m^−1^ data were evaluated at MQC (400 ng band^−1^) using their reported formulae [48]. Generally, the system efficiency is evaluated at single concentration. Among, LQC, MQC, and HQC, MQC is the most widely studied concentration for the evaluation of system suitability. As a consequence, the system suitability was determined at MQC in this work.

The accuracy of the greener SI-HPTLC test was determined using % recovery. At LQC (100 ng band^−1^), MQC (400 ng band^−1^), and HQC (1200 ng band^−1^), the percent recovery of TPM was measured.

The greener normal-phase SI-HPTLC method was given an intra/intermediate precision rating. The proposed analytical assay was used to examine intra-day variance by determining TPM at LQC, MQC, and HQC on the same day. The greener normal-phase SI-HPTLC approach was used to investigate inter-day precision by determining TPM at the same QC levels on three consecutive days [47].

To verify the robustness, a little change in the mobile phase for the greener SI-HPTLC method was made. For the robustness assessment, the initial CyHex-EtAc (40:60, *v v*^−1^) mobile phase was transformed to CyHex-EtAc (42:58, *v v*^−1^) and CyHex-EtAc (38:62, *v v*^−1^) mobile phases, with the necessary adjustments in densitometric response and R_f_ values stated [47]. Generally, the robustness is evaluated at single concentration. Among, LQC, MQC, and HQC, MQC is the most widely studied concentration for the evaluation of the robustness. As a consequence, the robustness was determined at MQC in this work.

Using a reported standard deviation technique, the sensitivity of the suggested analytical method was tested as “limit of detection (LOD) and limit of quantification (LOQ)”. The proposed analytical method’s “LOD and LOQ” were computed using their standard equations [47,48].

The peak purity/specificity was evaluated by comparing the R_f_ values and UV absorption spectra of TPM in marketed tablet dosage forms with those of pure TPM for the greener SI-HPTLC technique. The specificity of the method depends on the superimposed spectra of standard and test samples. For its evaluation, the drug peak is important instead of blank. Therefore, the blank sample was not used to study the specificity of the method.

### 2.6. Selectivity and Degradation Evaluations

The selectivity and degradation studies for the proposed analytical method were conducted under acid, base, oxidative, thermal, and photolytic degradation conditions [48,49]. For this evaluation, the MQC level of TPM (400 ng band^−1^) was exposed to 1 M HCl (acid), 1 M NaOH (base), 30% *v v*^−1^ H_2_O_2_ (oxidative), a hot air oven at 55 °C for 24 h (thermal), and a 254-nm light in a UV chamber for 24 h (photolytic). The procedure as reported in our recent publications was adopted for these studies [49]. TPM chromatograms were recorded and evaluated for degradation patterns under various stress settings.

### 2.7. Analysis of TPM in Marketed Tablets

The densitometric responses of the prepared solutions of marketed tablet dosage forms were recorded on TLC plates. The TPM amounts in marketed tablet dosage forms were calculated utilizing a TPM calibration plot for the proposed analytical method.

### 2.8. Greenness Evaluation

The AGREE metric methodology [41] was used to evaluate the greener profile of the suggested analytical method. “AGREE: The Analytical Greenness Calculator (version 0.5, Gdansk University of Technology, Gdansk, Poland, 2020)” was utilized to predict the AGREE scores (0.0–1.0) for the suggested analytical test.

## 3. Results and Discussion

### 3.1. Method Development

**Figure 1:**
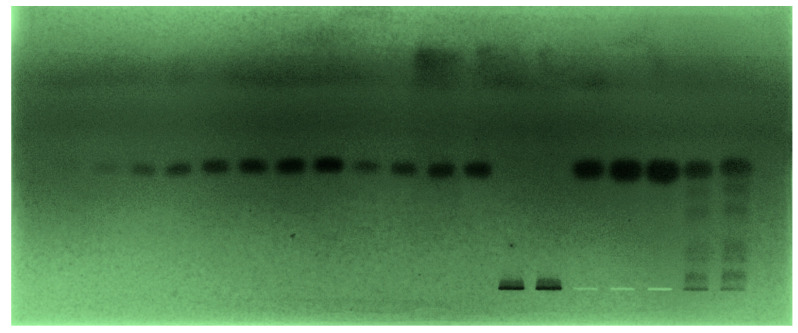
Thin-layer chromatography (TLC)-chromoplate of standard topiramate (TPM) and commercial tablet dosage forms established using CyHex-EtAc (40:60, *v v*^−1^) as the greener mobile phase system for the greener normal-phase high-performance thin-layer chromatography (HPTLC) method.

**Figure 2:**
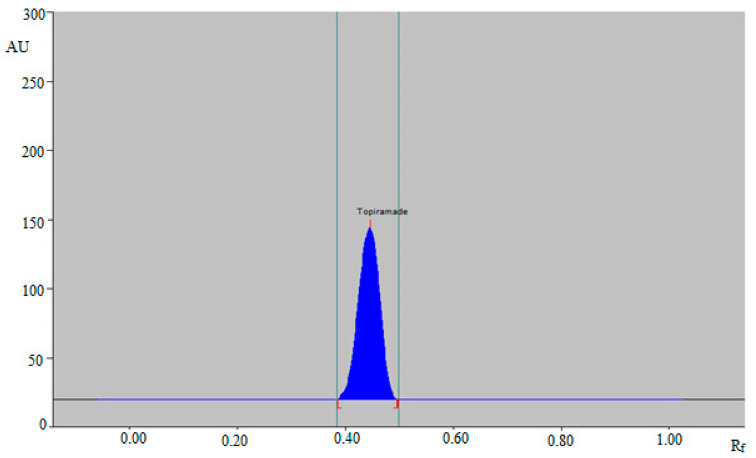
HPTLC chromatogram of 400 ng band^−1^ concentration of pure TPM for the suggested HPTLC assay.

Despite the fact that various pharmaceutical assays for TPM measurement have been documented, there are no greener HPTLC techniques for TPM quantitation in the literature. As a consequence, the objective of the current study is to design a greener SI-HPTLC technique for TPM analysis in commercial tablet dosage forms. Different quantities of CyHex and EtAc were tested as the greener mobile phases for the formation of a suitable band for TPM analysis, such as CyHex-EtAc (40:60, *v v*^−1^), CyHex-EtAc (50:50, *v v*^−1^), CyHex-EtAc (60:40, *v v*^−1^), CyHex-EtAc (70:30, *v v*^−1^), and CyHex-EtAc (80:20, *v v*^−1^). The chamber saturation conditions were used to develop all greener mobile phases (Figure 1). The data revealed that the greener mobile phases of CyHex-EtAc (50:50, *v v*^−1^), CyHex-EtAc (60:40, *v v*^−1^), CyHex-EtAc (70:30, *v v*^−1^), and CyHex-EtAc (80:20, *v v*^−1^) had a poor TPM densitogram with an unacceptable As value (As = 1.24). The greener CyHex-EtAc (40:60, *v v*^−1^) mobile phase, on the other hand, showed a well-resolved TPM peak at R_f_ = 0.45 ± 0.01 with a reliable As value (As = 1.02 ± 0.02) (Figure 2). As a consequence, the greener mobile phase for TPM measurement in its marketed formulations was chosen as CyHex-EtAc (40:60, *v v*^−1^). The different peak parameters using a different composition of mobile phase could be due to differences in the polarity of mobile phases studied. The UV-spectral bands for the greener normal-phase SI-HPTLC method were determined at densitometric mode, and the maximum TLC response was observed at 423 nm after derivatization with anisaldehyde-sulfuric acid for the greener SI-HPTLC method. Hence, the entire quantification of TPM was carried out at 423 nm.

### 3.2. Validation Studies

For several parameters, the suggested analytical approach for TPM analysis was validated [47]. The linearity study of the TPM calibration plot for the suggested analytical method is documented in Table 1. The TPM calibration plot for the proposed analytical approach was linear in the 30–1200 ng band^−1^ range. For the greener SI-HPTLC method, the determination coefficient (R^2^) and regression coefficient (R) for TPM were determined to be 0.9945 and 0.9973, respectively. TPM content and TLC response had a good linear connection according to these findings.

At MQC (400 ng band^−1^), the parameters for the greener SI-HPTLC method’s system suitability/efficiency were examined, and the results are presented in Table 2. The Rf, As, and N m^−1^ values for the greener SI-HPTLC method were 0.45 ± 0.01, 1.02 ± 0.02, and 4780 ± 3.11, respectively. These results demonstrated that the suggested analytical method may be used to quantify TPM in the marketed tablets.

The results of the accuracy evaluation for the greener SI-HPTLC method are summarized in Table 3. The percent TPM recovery for the suggested SI-HPTLC test was calculated to be between 98.43 and 101.08% at three different QC levels. The accuracy of the suggested analytical approach for TPM analysis in its marketed formulations was demonstrated by these percent TPM recoveries.

The precision for the suggested SI-HPTLC method was determined as the percent of the coefficient of variation (% CV), and the results are documented in Table 4. The % CVs of TPM for the suggested SI-HPTLC method were recorded as 0.47–0.92% at three different QC levels for the intra-day variation. The % CVs of TPM for the suggested SI-HPTLC method were recorded as 0.48–0.97% at three different QC levels for the inter-day variation. These results suggest the precision of the suggested SI-HPTLC method for TPM analysis in its marketed tablet dosage forms.

The results of the robustness assessment for the greener SI-HPTLC test are summarized in Table 5. The % CVs for the robustness assessment for the greener SI-HPTLC technique were estimated to be 0.73–0.77%. The R_f_ values were found to be in the range of 0.44–0.46. The robustness of the suggested analytical approach for TPM analysis in its marketed tablet dosage forms was demonstrated by minimal variations in TPM R_f_ values and low percent CVs.

The suggested analytical method’s sensitivity was tested as “LOD and LOQ”, and their projected values are listed in Table 1. For TPM analysis, the “LOD and LOQ” for the suggested analytical technique were 10.16 ± 0.21 and 30.48 ± 0.63 ng band^−1^, respectively. The sensitivity for TPM analysis in its marketed tablets was demonstrated by these “LOD and LOQ” figures for the greener SI-HPTLC method.

**Figure 3:**
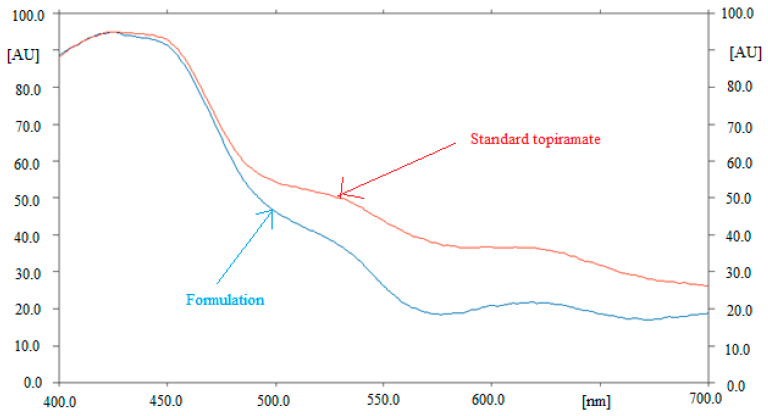
UV-absorption spectra of pure TPM and marketed tablets, superimposed.

The peak purity/specificity of the proposed analytical approach was determined by comparing the superimposed UV-absorption spectra of TPM in commercial tablet dosage forms with those of pure TPM. Figure 3 compares the UV absorption spectra of pure TPM with those of TPM in marketed tablet dosage forms. After derivatization in visible mode, the greatest TLC response for TPM in pure form and commercial tablets was obtained at 423 nm. The same UV-absorption spectra, R_f_ values, and detection wavelength of TPM in pure TPM and marketed tablet dosage forms confirmed the peak purity/specificity for the suggested SI-HPTLC method.

### 3.3. Selectivity and Degradation Studies

**Figure 4:**
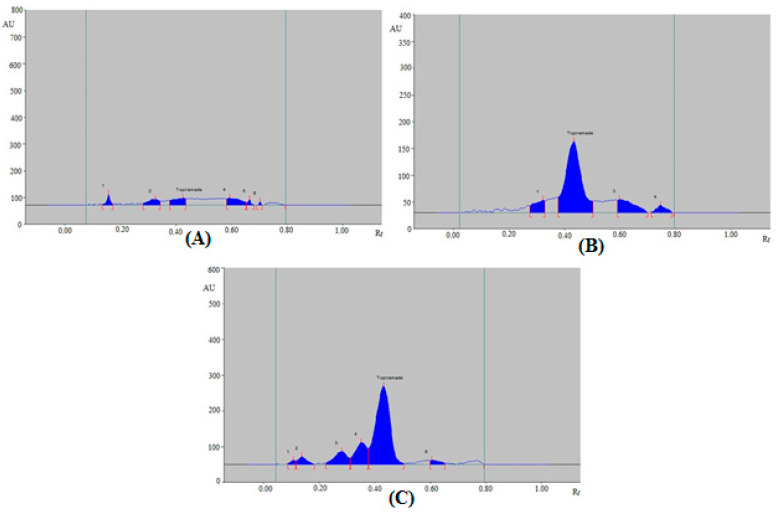
Representative HPTLC chromatograms of TPM recorded under (**A**) acid-induced degradation, (**B**) base-induced degradation, and (**C**) oxidative degradation of TPM.

Under various stress circumstances, the selectivity and degradation of the greener normal-phase SI-HPTLC technique were investigated. Figure 4 and Table 6 summarize the results of the greener normal-phase SI-HPTLC approach. TPM peaks were well-separated in chromatographic peaks from degradation experiments, with some extra peaks of degradation products (Figure 4). Under acid-degradation conditions, 28.66% of TPM remained intact, while 71.34% decomposed (Table 6 and Figure 4A). Therefore, TPM was sufficiently degraded under acid-degradation condition. The acid-induced degradation peaks (peaks 1, 2, 4, 5, and 6 in Figure 4A) were resolved with R_f_ values of 0.16, 0.33, 0.59, 0.66, and 0.70, respectively. The R_f_ value of TPM under acid-degradation condition was slightly shifted (R_f_ = 0.44). TPM remained at 71.99% in the base-degradation scenario, while 28.01% was degraded (Table 6 and Figure 4B). The base-induced degradation peaks (peaks 1, 3, and 4 in Figure 4B) were resolved with R_f_ values of 0.32, 0.66, and 0.75, respectively. The R_f_ value of TPM under base-degradation condition was also slightly shifted (R_f_ = 0.44). 64.53% of TPM remained after oxidative degradation while 35.47% was degraded (Table 6 and Figure 4C). The H_2_O_2_-induced degradation peaks (peaks 1, 2, 3, 4, and 6 in Figure 4C) were resolved with R_f_ values of 0.11, 0.14, 0.28, 0.35, and 0.61, respectively. The R_f_ value of TPM under oxidative-degradation condition was also slightly shifted (R_f_ = 0.44). TPM maintained at 100.00% during thermal and photolytic degradation conditions (figure not shown), and no degradation was observed. As a result, TPM was extremely resistant to thermal and photolytic degradation. Under acid-degradation condition, the maximal degradation of TPM was measured using the greener normal-phase SI-HPTLC method. All of these findings suggest that TPM may be detected in the presence of its degradation products using the greener normal-phase SI-HPTLC approach. The selectivity and stability-indicating property of the greener normal-phase SI-HPTLC method are proposed by these results and observations.

### 3.4. Determination of TPM in Commercial Tablet Dosage Forms

The suggested analytical method was used to determine TPM in commercial tablet dosage forms based on acceptable validation parameters. By comparing its TLC band at R_f_ = 0.45 ± 0.01 with those of pure TPM for the greener normal-phase SI-HPTLC method, the chromatographic peak of TPM from commercial tablet dosage forms was discovered. TPM’s chromatographic peak in commercial tablet dosage forms was also found to have R_f_ = 0.45 ± 0.01. The greener normal-phase SI-HPTLC method was selective for TPM without interference from the other ingredients of the tablets. The calibration plot of TPM was used to calculate the TPM content of commercial tablets. TPM concentration in commercial tablet dosage form was found to be 99.14%. These results showed that the proposed analytical method can be utilized for TPM analysis in its pharmaceutical products.

### 3.5. Greenness Evaluation

**Figure 5:**
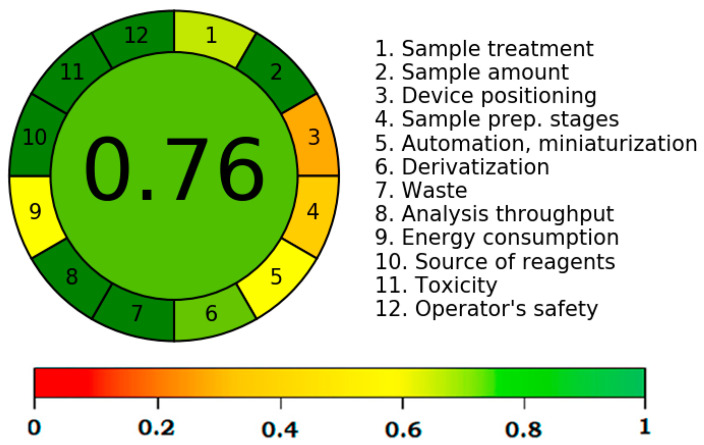
Analytical GREEnness (AGREE) score for the suggested HPTLC assay.

**Figure 6:**
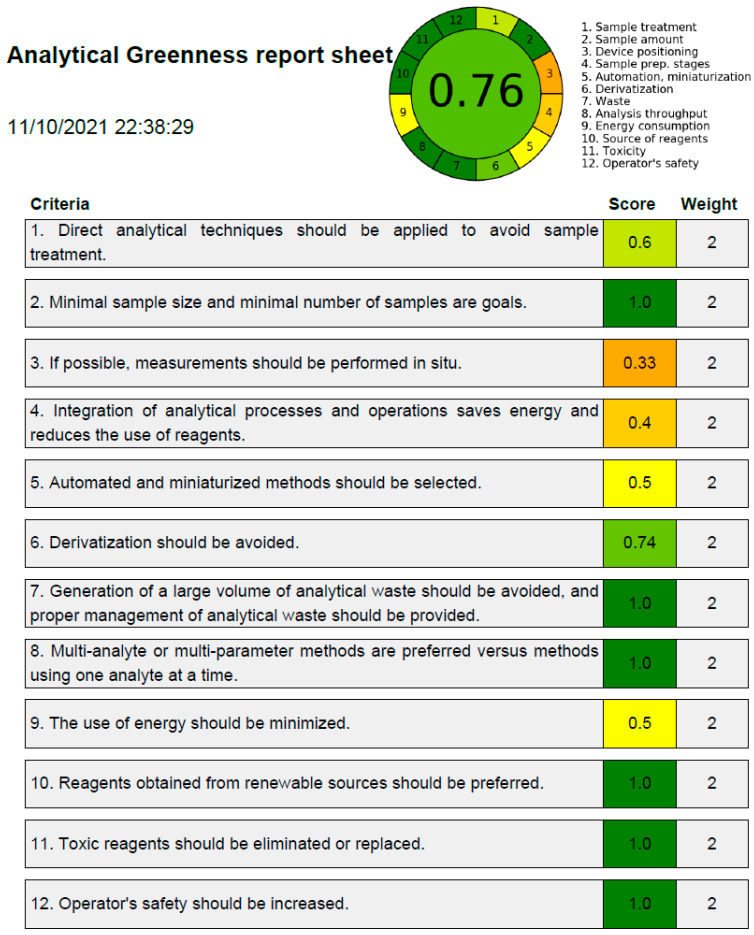
AGREE scale sheet for the suggested HPTLC method of TPM, indicating the AGREE score for 12 different GAC principles.

Despite the fact that there are various ways in the literature to forecast greener profiles of analytical procedures [37,38,39,40,41,42], only the AGREE methodology [41] incorporates all 12 GAC principles. Accordingly, the suggested analytical method’s greenness was assessed using the AGREE Calculator. The overall AGREE scale for the suggested analytical method is depicted in Figure 5. The AGREE report sheets for each GAC component/principle are summarized in Figure 6. The suggested analytical method received an overall AGREE score of 0.76, suggesting that the suggested strategy for TPM analysis is extremely green.

### 3.6. Comparison with Reported Methods

The greener HPTLC method for TPM analysis in pharmaceutical formulations was compared with reported analytical techniques. Table 7 displays the comparative results.

Three different parameters, including linearity range, accuracy, and precision of the greener HPTLC method, were compared. The linearity for the reported colorimetry and flow-injection spectrometry methods was found to be inferior to the greener HPTLC method [6,19]. However, the accuracy and precision of the of reported colorimetry and flow-injection spectrometry methods were within the limits of ICH guidelines and thus similar to the greener HPTLC method [6,19]. The linearity range of various HPLC methods was also inferior to the greener HPLTLC method [4,9,10,11]. However, the accuracy and precision of these HPLC methods were similar to the greener HPTLC method [4,9,10,11]. The linearity range and precision for another HPLC method were also inferior to the greener HPTLC method [8]. The linearity range of reported LC-MS method was superior to the greener HPTLC method [17]. However, its accuracy was inferior to the greener HPTLC method [17]. The linearity of reported UPLC and NMR methods was also inferior to the greener HPTLC method [18,20], while the accuracy and precision of these methods were similar to the greener HPTLC method [18,20]. The linearity, accuracy, and precision of reported HPTLC methods was also inferior to the greener HPTLC method [12,14]. Based on all these observations and results, the greener HPTLC method was found to be reliable and superior over reported analytical techniques of TPM analysis in pharmaceutical formulations.

## 4. Conclusions

The goal of this project was to design and validate a normal-phase SI-HPTLC method for determining TPM in tablet dosage forms that is rapid, more sensitive, and more environmentally friendly. The greener SI-HPTLC method was validated as per ICH recommendations. The greener SI-HPTLC method was sensitive, rapid, greener, and selective for TPM analysis. The overall AGREE score for the greener SI-HPTLC method indicated its excellent greener profile for TPM analysis. The greener SI-HPTLC method was able to detect TPM along with its degradation products, suggesting the selectivity and stability-indication property of the suggested method. In its marketed tablet dosage forms, the greener SI-HPTLC method was reliable for TPM analysis. These findings suggest that the SI-HPTLC approach, which is more environmentally friendly, might be used for TPM analysis in pharmaceutical products.

## Figures and Tables

**Table 1:** Results of the linearity study for topiramate (TPM) analysis using the greener high-performance thin-layer chromatography (HPTLC) method ^a^.

Parameters	Values ^a^
Linearity range (ng band^−1^)	30–1200
Regression equation	y = 7.5498x + 524.24
R^2^	0.9945
R	0.9973
Slope ± SD	7.5498 ± 0.32000
Intercept ± SD	524.24 ± 7.2800
Standard error of slope	0.130
Standard error of intercept	2.97
95% confidence interval of slope	6.9875–8.1120
95% confidence interval of intercept	511.44–537.03
LOD ± SD (ng band^−1^)	10.16 ± 0.21
LOQ ± SD (ng band^−1^)	30.48 ± 0.63

^a^ Mean ± SD; n = 6; LOD: limit of detection; LOQ: limit of quantification.

**Table 2:** The parameters for system suitability/efficiency of TPM for the suggested HPTLC method.

Conc. (ng band^−1^)	Parameters	Value ^a^
	R_f_	0.45 ± 0.01
400	As	1.02 ± 0.02
	N m^−1^	4780 ± 3.11

^a^ Mean ± SD; n = 3; R_f_: retardation factor; As: asymmetry factor; N m^−1^: theoretical plates number.

**Table 3:** The % TPM recoveries for the suggested HPTLC method ^a^.

Conc. (ng band^−1^)	Conc. Found (ng band^−1^) ± SD	Recovery (%)	CV (%)
100	101.08 ± 1.41	101.08	1.39
400	395.68 ± 2.41	98.92	0.60
1200	1181.24 ± 5.31	98.43	0.44

^a^ Mean ± SD; n = 6.

**Table 4:** Determination of TPM precision for the suggested HPTLC method ^a^.

Conc. (ng band^−1^)	Intra-Day Precision			Inter-Day Precision		
	Conc. (ng band^−1^) ± SD	Standard Error	CV (%)	Conc. (ng band^−1^) ± SD	Standard Error	CV (%)
100	99.11 ± 0.92	0.37	0.92	100.25 ± 0.98	0.40	0.97
400	403.54 ± 2.52	1.02	0.62	396.21 ± 2.71	1.10	0.68
1200	1187.23 ± 5.61	2.29	0.47	1210.32 ± 5.84	2.38	0.48

^a^ Mean ± SD; n = 6.

**Table 5:** Results of robustness assessment of TPM for the greener normal-phase HPTLC method ^a^.

Conc. (ng band^−1^)	Mobile Phase Composition (CyHex-EtAc)			Results		
	Original	Used	Level	Conc. (ng Band^−1^) ± SD	% CV	R_f_
		42:58	+2.0	393.65 ± 2.89	0.73	0.44
400	40:60	40:60	0.0	404.23 ± 3.02	0.74	0.45
		38:62	−2.0	408.21 ± 3.15	0.77	0.46

^a^ Mean ± SD; n = 6.

**Table 6:** Results of forced-degradation studies of TPM at different stress conditions for the greener normal-phase HPTLC method ^a^.

Stress Condition	Number of Degradation Products (R_f_)	TPM R_f_	TPM Remaining (ng band^−1^)	TPM Recovered (%)
1 M HCl	5 (0.16, 0.33, 0.59, 0.66, 0.70)	0.44	114.24	28.56 ± 1.41
1 M NaOH	3 (0.32, 0.66, 0.75)	0.44	287.96	71.99 ± 1.78
30% H_2_O_2_	5 (0.11, 0.14, 0.28, 0.35, 0.61)	0.44	258.12	64.53 ± 1.57
Photolytic	0	0.45	400.00	100 ± 0.00
Thermal	0	0.45	400.00	100 ± 0.00

^a^ Mean ± SD; n = 3.

**Table 7:** Comparison of the suggested HPTLC approach with reported methods of TPM analysis in pharmaceutical formulations.

Analytical Method	Linearity Range	Accuracy (% Recovery)	Precision (% CV)	Ref.
Colorimetry	100–1200 (µg mL^−1^)	99.97–100.02	0.54–0.58	[6]
Flow injection spectrometry	5–35 (µg mL^−1^)	99.70–101.30	1.30–2.00	[19]
HPLC	1–300 (µg mL^−1^)	-	1.35–1.45	[4]
HPLC	1–100 (µg mL^−1^)	99.93	0.35–3.23	[8]
HPLC	5000–15,000 (µg mL^−1^)	-	0.30–1.00	[9]
HPLC	10–50 (µg mL^−1^)	100.02–100.57	0.08–0.19	[10]
HPLC	50–3000 (µg mL^−1^)	99.60	0.17–0.65	[11]
LC-MS	1–1000 (ng mL^−1^)	93.30–99.70	0.10–1.85	[17]
UPLC	50–150 (µg mL^−1^)	99.00–99.70	0.05–0.10	[18]
NMR	50–850 (µg mL^−1^)	98.86–99.70	<2.00	[20]
HPTLC	1000–5000 (ng band^−1^)	89.11–102.24	3.10–5.16	[12]
HPTLC	250–4000 (ng band^−1^)	104.47	4.16	[14]
HPTLC	30–1200 (ng band^−1^)	98.43–101.08	0.47–0.97	Present work

## Data Availability

Not applicable.
